# Significance of the thrombo-inflammatory status-based novel prognostic score as a useful predictor for in-hospital mortality of patients with type B acute aortic dissection

**DOI:** 10.18632/oncotarget.18105

**Published:** 2017-05-23

**Authors:** Dongze Li, Lei Ye, Jing Yu, Lixia Deng, Lianjing Liang, Yan Ma, Lei Yi, Zhi Zeng, Yu Cao, Zhi Wan

**Affiliations:** ^1^ Department of Emergency Medicine, West China Hospital, Sichuan University, Chengdu, China; ^2^ Department of Cardiology, West China Hospital, Sichuan University, Chengdu, China; ^3^ School of Public Health, Xinjiang Medical University, Urumqi, China; ^4^ Department of Neurology, First Affiliated Hospital of Xinjiang Medical University, Urumqi, China

**Keywords:** acute aortic dissection, inflammation, thrombosis, prognosis

## Abstract

**Background:**

Inflammation and thrombosis are associated with development and progression of acute aortic dissection (AAD). The aim of this study was to assess the prognostic significance of Simplified Thrombo-Inflammatory Prognostic Score (sTIPS), in patients with early phase type B AAD.

**Methods:**

We retrospectively reviewed 491 patients with type B AAD between November 2012 and September 2015. sTIPS was calculated from the white blood cell count (WBC) and mean platelet volume to platelet count (MPV/PC) ratio, at the time of admission. Patients with both, WBC > 10 (10^9^/L) and MPV/PC ratio > 7.5 (10^2^fL/10^9^L^-1^) were assigned a score of 2, while patients with high levels of either one or none of the above markers, were assigned scores of 1 and 0 respectively. Multivariable Cox regression analyses were used to investigate the associations between the score and hospital survival.

**Results:**

Of the 491 type B AAD patients included in this analysis, 24 patients (4.9%) died during hospitalization. Kaplan-Meier analysis revealed that the cumulative mortality was significantly higher in patients with higher sTIPS (*P* = 0.001). Multivariable Cox regression analysis further indicated that higher sTIPS was a strong predictor of in-hospital mortality after eliminating all confounding factors (sTIPS 2: hazard ratio 4.704, 95%; confidence interval [CI] 1.184-18.685; *P* = 0.028; sTIPS 1: hazard ratio 1.918, 95%; CI 1.134-3.537; *P* = 0.045).

**Conclusions:**

sTIPS at admission was a useful tool for stratifying the risk in type B AAD patients, for outcomes such as in-hospital mortality in the early phase.

## INTRODUCTION

Type B acute aortic dissection (AAD) is a life-threatening emergency, which is associated with high morbidity and mortality rates [1]. Despite clinical advancements to aid in early diagnosis, as well as surgical and endovascular therapies, the mortality rate for type B AAD in the acute phase remains high. Data from the International Registry of Acute Aortic Dissection (IRAD) indicate that in-hospital mortality rate of patients with type B AAD was 9.2%, and it was similar among patients undergoing endovascular repair and those who are medically managed [2]. Therefore, predictive tools that can identify patients with type B AAD at increased risk of death in the acute phase are critical for risk stratification and treatment decisions. Some clinical prognostic markers for AAD have been identified, including old age [2], female sex [3], hypotension [2], impaired renal function [4], and hypoalbuminemia [5]. Some others markers, such as maximum aortic diameter and false lumen closure status, are evaluated using computed tomography (CT) [6, 7]. However, the anatomy of aortic dissection is often complex and might not be consistent, especially during the early hospital stay. It is therefore challenging to evaluate the severity of AAD from these parameters alone, in the early phase of admission.

Tolenaar and colleagues attempted to design a simple prediction model using variables that are independently associated with in-hospital mortality in patients with type B AAD, including age, hypotension/shock, periaortic hematoma, descending aorta diameter, mesenteric ischemia, acute renal failure, and limb ischemia [2]. However, it is challenging for physicians to identify high-risk patients with type B AAD in the early phase, especially in the emergency room. Inflammatory response and thrombosis play an important role in the progression of AAD; thrombo-inflammatory status is associated with the severity and type of AAD, and correlates with adverse outcomes [8, 9]. Our previous study indicated that a novel prognostic score based on thrombo-inflammatory status (Simplified Thrombo-Inflammatory Prognostic Score, sTIPS) was a useful tool for stratifying high-risk patients with type A AAD, irrespective of surgical or medical treatment [10]. However, it was unclear whether sTIPS could also predict mortality in patients with type B AAD; we thus conducted the present study to investigate the association between sTIPS and the risk of in-hospital mortality in patients with type B AAD.

## MATERIALS AND METHODS

This was a single-center, retrospective cohort study, to evaluate the association between sTIPS and in-hospital

mortality risk, in patients with type B AAD. The study complied with the Declaration of Helsinki, and the study protocol was approved by the Human Ethical Committee of West China Hospital of Sichuan University.

### Patient selection and endpoints

We retrospectively reviewed hospital records between November 2012 and September 2015, at West China Hospital of Sichuan University. Records of all hospitalized patients diagnosed with type B AAD were screened. The inclusion criteria were as follows: type B AAD presenting within 14 days of onset of symptoms, and dissection of descending aorta with presence of both, true and false lumen confirmed on CT with contrast. The exclusion criteria were, patients with malignant tumor (three patients), infectious diseases (nine patients), history of chronic liver disease (five patients), and splenomegaly (two patients). Additionally, 18 patients were excluded because the auxiliary examination could not be conducted. Finally, 491 patients with type B AAD were enrolled in this study. The primary endpoint was all-cause mortality.

### Data collection

Clinical data of each patient was extracted from clinical notes and charts in the hospital. These included demographic data, cardiovascular risk factors, past medical history, results of blood tests at admission, electrocardiographic and CT findings, details of therapies, drug treatments during the hospital stay and at discharge, and occurrence of in-hospital death. The Modification of Diet in Renal Disease equation was used to estimate glomerular filtration rate in milliliters per minute per 1.73 m^2^ [11].

### Simplified thrombo-inflammatory prognostic score

sTIPS is described in our previous study [10]; in the present study, it was calculated from data obtained on the day of admission. Patients with both, white blood cell count (WBC) > 10 (10^9^/L) and a mean platelet volume to platelet count (MPV/PC) ratio > 7.5 (10^2^fL/10^9^L^-1^) were assigned a score of 2, while patients showing elevations in either only one or none of the parameters, were assigned scores of 1 and 0, respectively.

### Statistical analysis

Data were calculated as frequencies and percentages for categorical variables, and as mean ±SD for continuous variables (normal distribution) or median and interquartile ranges (abnormal distribution). Patient characteristics were compared based on sTIPS values. Parametric patient characteristics were compared using a one-way ANOVA, whereas nonparametric characteristics were compared using the Kruskal-Wallis H test. Categorical data were compared using the chi-square (χ^2^) test. Kaplan-Meier curves were constructed and stratified according to sTIPS. Cox proportional hazards models were used to investigate the relationship between sTIPS and time-to-mortality during hospitalization. To construct the Cox model, univariate Cox regression for each predicting variable was performed, with all-cause mortality as the outcome variable. Variables that were found to be significant (*P* < 0.05) on univariate Cox models were then entered into a multivariable Cox model. From the multivariable model, we identified variables that were significant (*P* < 0.05) predictors of mortality. Data analysis was performed using SPSS Statistics for Windows, Version 21.0 (Chicago, IL, USA: SPSS Inc.).

## RESULTS

### Baseline patient characteristics

In this study, 491 patients with type B AAD were enrolled to investigate the association between sTIPS and the risk of in-hospital death. Of 491 patients, 24 patients (4.9%) died during hospitalization. Of these, 16 patients died of rupture of the aortic dissection, 4 of myocardial infarction, and 2 each of shock and acute kidney failure. The mean age of patients was 53 ± 13 years; 425 patients (86.6%) were male, 135 patients (27.5%) received medication, 337 patients (68.6%) received endovascular therapy, and 19 patients (3.9%) underwent surgery. Of these, 17 (12.6%), 6 (1.8%), and 1 (5.3%) on medication, endovascular therapy, and surgery, respectively, died during hospitalization. The median time of presentation from onset of symptoms was 0.98 (0.5-1.9) days. The median duration of hospital stay was 14 (3-20) days.

Baseline characteristics of the study cohort classified by sTIPS, are shown in Table 1. Age, WBC, MPV/PC ratio, albumin level, uric acid level, and prevalence of hypertension and smoking were significantly different among the three sTIPS groups. Other clinical characteristics did not vary significantly by sTIPS (Table 1).

**Table 1:** Relationships between clinical characteristics and the sTIPS in patients with type B acute aortic dissection.

Variables	sTIPS0 (*n*=194)	sTIPS1 (*n*=228)	sTIPS2 (*n*=69)	*P*- value
Age (years)	54±13	51±12	53±13	0.021
Males, *n* (%)	160 (82.5)	203 (89.0)	62 (89.9)	0.099
Hypertension, n (%)	161 (83.0)	166 (72.8)	57 (82.6)	0.026
Coronary artery disease, *n* (%)	5 (2.6)	7 (3.1)	3 (4.3)	0.746
Marfan syndrome, *n* (%)	1 (5.2)	2 (8.8)	1 (1.4)	0.752
Diabetes mellitus, *n* (%)	27 (13.9)	21 (9.2)	10 (14.5)	0.249
Smoking, *n* (%)	86 (44.3)	122 (53.5)	42 (60.9)	0.035
Admission SBP (mmHg)	145±26	151±28	158±27	0.001
Admission DBP (mmHg)	87±17	90±20	93±19	0.065
Admission heart rate (beats/min)	82±14	86±15	86±17	0.009
**Blood parameters**				
WBC (109/L)	7.3±1.8	12.1±4.2	13.0±3.1	<0.001
Platelet count(109/L)	212(168-274)	177(135-227)	125(112-136)	<0.001
MPV (fL)	10(9.1-10.9)	10.9(9.8-12.0)	12.2(10.9-13.2)	<0.001
MPV/PC ratio (102fL/109L-1)	4.1±2.1	6.3±3.6	10.5±3.3	<0.001
Hemoglobin (g/L)	122±22	129±23	130±23	0.004
Creatinine (umol/L)	78(60-93)	84(71-102)	82(65-103)	0.015
eGFR (mL/min)	100(65-132)	119(88-161)	117(78-154)	0.001
Urea nitrogen (mmol/L)	6.3±3.7	7.5±6.9	6.9±3.8	0.102
Albumin (g/L)	34±6	36±6	36±5	0.010
Uric acid (mmol/L)	289(211-346)	311(242-415)	294(231-348)	0.003
TC (mmol/L)	2.60±1.78	2.67±1.58	2.82±1.56	0.667
TG (mmol/L)	2.67±1.48	4.97±1.58	2.36±1.46	0.339
HDL-C (mmol/L)	0.87(0.64-1.11)	1.01(0.72-1.30)	1.06(0.85-1.46)	0.002
LDL-C (mmol/L)	2.27±0.70	2.20±0.72	2.19±0.69	0.595
Direct bilirubin (umol/L)	3.18(2.15-5.20)	3.90(2.08-5.80)	5.70(3.00-7.79)	<0.001
Indirect bilirubin (umol/L)	11.2±7.2	13.5±9.1	15.4±8.2	<0.001
Total bilirubin (umol/L)	15.7±8.9	19.4±11.4	21.6±9.6	<0.001
AST (IU/L)	19(16-29)	23(18-34)	25(19-37)	0.007
Lactate dehydrogenase (IU/L)	158(139-185)	178(154-220)	185(161-259)	<0.001
Creatine kinase (IU/L)	53(34-76)	82(43-184)	80(43-219)	<0.001
CK-MB (IU/L)	5.55(1.74-8.50)	6.30(1.58-11.15)	4.90(1.58-10.0)	0.080
LVEF (%)	62±7	62±9	63±7	0.596
**Types of treatment, *n* (%)**				**0.957**
Endovascular therapy	135 (69.6)	153 (67.1)	49 (71.0)	
Surgery	7 (3.6)	9 (4.0)	3 (4.4)	
Medication	52 (26.8)	66 (28.9)	17 (24.6)	
**Medication in hospital**				
ARB/ACEI, *n* (%)	94 (48.5)	98 (43.0)	37 (53.6)	0.242
CCB, *n* (%)	165 (85.1)	185 (81.1)	52 (75.2)	0.185
Beta-blockers, *n* (%)	173 (89.2)	214 (93.9)	64 (92.8)	0.206
Diuretics, *n* (%)	49 (25.3)	73 (32.0)	25 (35.6)	0.140
Sodium nitroprusside, *n* (%)	113 (58.2)	119 (52.2)	43 (62.2)	0.239

MPV, mean platelet volume; eGFR, estimated glomerular fltration rate; TC, total cholesterol; TG, total glyceride; HDL-C, high density lipoprotein cholesterol; LDL-C, low density lipoprotein cholesterol; AST, aspertate aminotransferase; CK-MB, creatine kinase isoenzyme; sTIPS, the Simplified Thrombo-Inflammatory Prognostic Score; SBP, systolic blood pressure at admission; DBP, diastolic blood pressure at admission; LVEF, left ventricular ejection fraction; WBC, white blood cell; MPV/PC ratio, mean platelet volume to platelet count ratio; ARB, angiotensin II receptor; ACEI, angiotensin-converting enzyme inhibitor; CCB, calcium-channel blocker

### STIPS and in-hospital mortality

**Figure 1:**
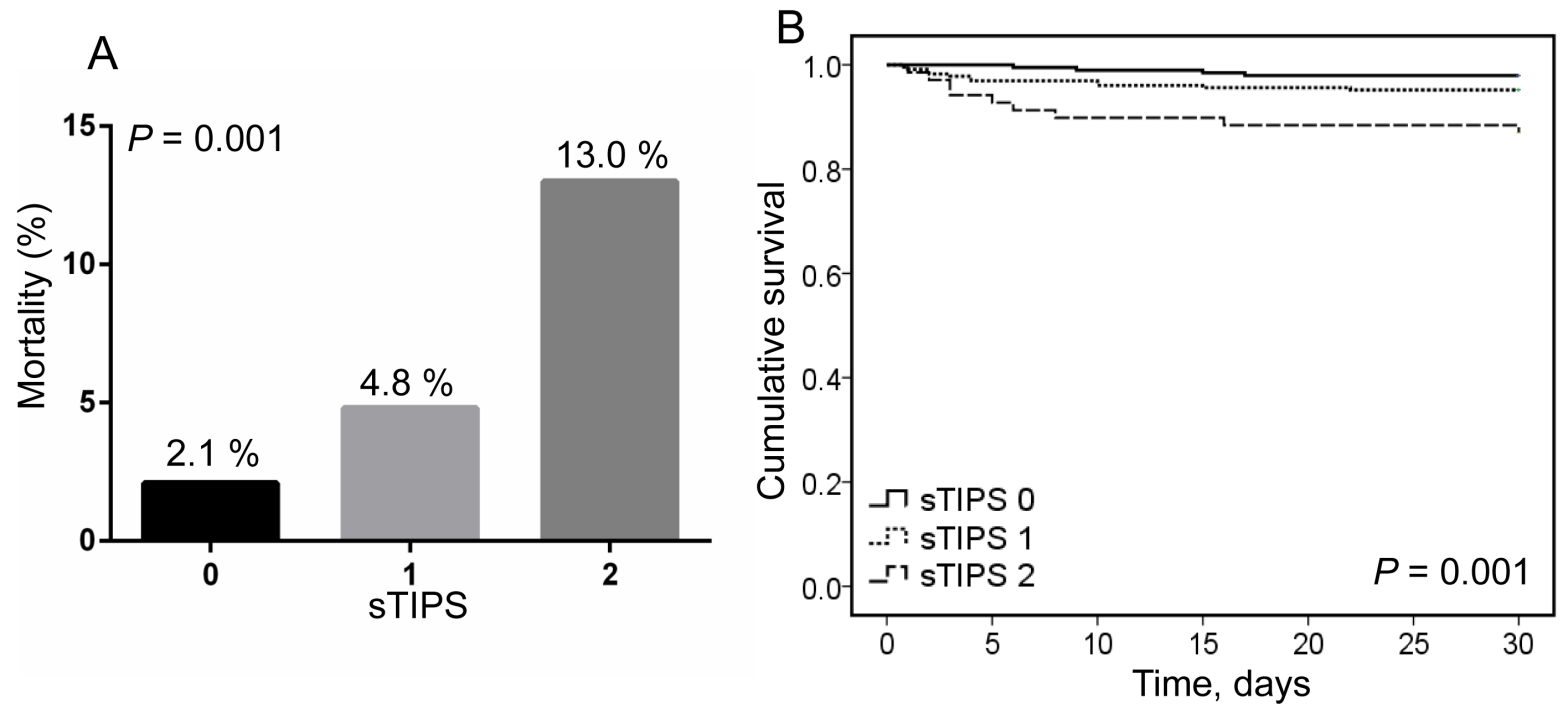
A. the short-term mortality in different thrombo-inflammatory prognostic score (sTIPS) patients with type B acute aortic dissection; B. Kaplan-Meier analysis survival curve according to different sTIPS in patients with type B acute aortic dissection (0 score: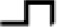, 1 score: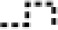, 2 score: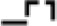)

**Figure 2:**
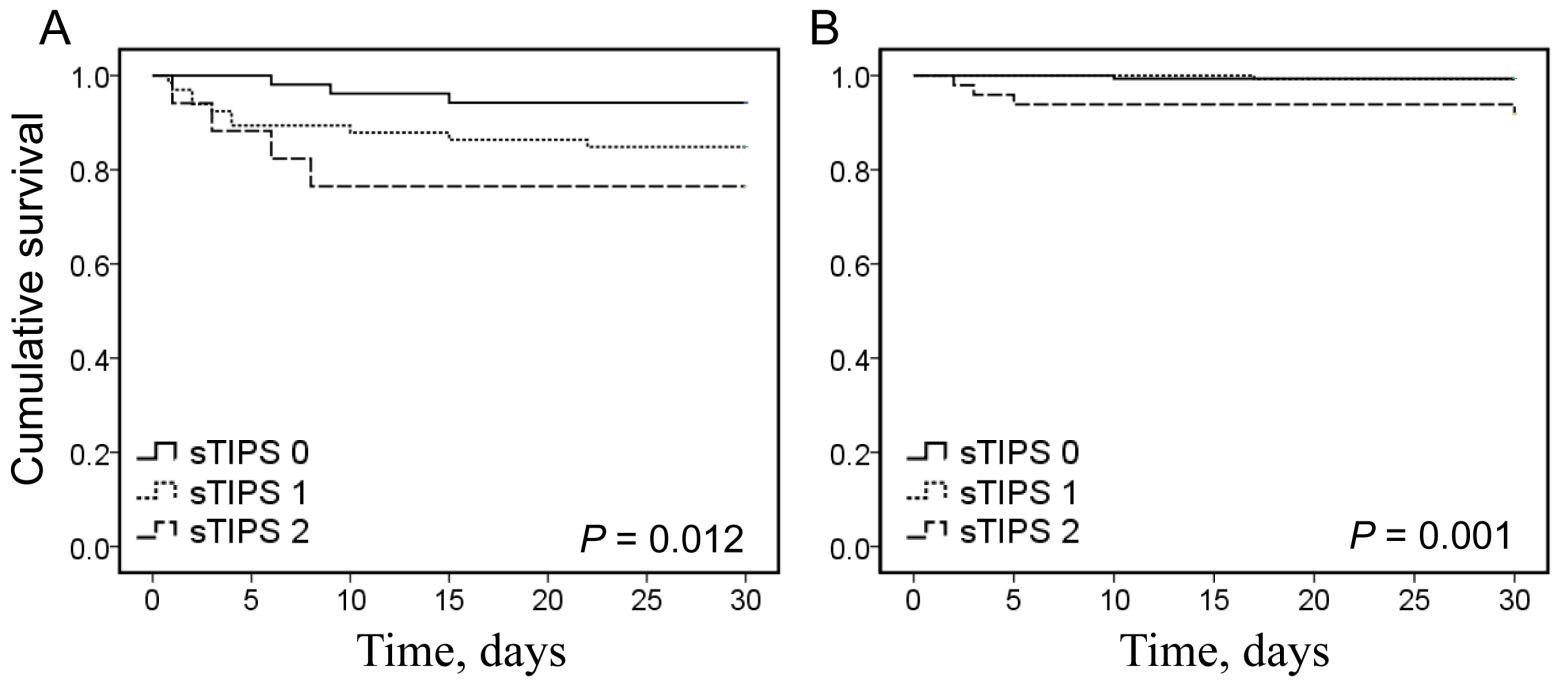
A. Kaplan-Meier analysis survival curve according to different thrombo-inflammatory prognostic score (sTIPS) in patients with type B acute aortic dissection receiving medication therapy **B.** Kaplan-Meier analysis survival curve according to different sTIPS in patients with type B acute aortic dissection receiving endovascular therapy. (0 score: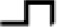, 1 score: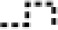, 2 score: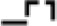).

The in-hospital mortality rate of patients gradually increased as sTIPS increased; the mortality rate of patients with a score of 2 was 2.7 and 6.2 times higher than that in patients with scores of 1 or 0, respectively (Figure 1A). Kaplan-Meier survival analysis showed that the cumulative mortality rate was significantly greater in patients with higher sTIPS (*P* = 0.001; Figure 1B). Subgroup analysis by treatment type indicated that the cumulative survival rate was lower in patients with higher sTIPS in the medication only group (*P* = 0.012; Figure 2A), as well as the endovascular therapy group (*P* = 0.001; Figure 2B). We did not analyze the relationship between score and in-hospital mortality of patients undergoing surgery as only one patient in this group died in the hospital.

Univariate Cox survival regression showed that sTIPS was positively related with the risk of all-cause mortality. Other variables that were significant in univariate Cox models are shown in Table 2. After adjusting for these potential confounders, the multivariable Cox survival regression indicated that high sTIPS continued to show a positive association with the risk of mortality. For sTIPS of 0, 1, and 2, the adjusted hazard ratios (HRs) [95% confidence intervals (95% CI)] for in-hospital mortality were 1.0 (ref.), 1.918 (95% CI: 1.134-3.537) (*P* = 0.045), and 4.704 (95% CI: 1.184-18.685) (*P* = 0.028), respectively (Table 2).

**Table 2:** Cox regression of short-term all-cause mortality for patients with type B acute aortic dissection.

Variables	Univariate analysis			Multivariate analysis		
	HR	95% CI	*P*	HR	95% CI	*P*
STIPS			0.002			0.016
STIPS1 (*vs*. STIPS0)	2.389	1.761-7.501	0.036	1.918	1.134-3.537	0.043
STIPS2 (*vs*. STIPS0)	6.717	2.068-21.813	0.002	4.704	1.184-18.685	0.028
WBC >10 (109/L)	3.414	1.355-8.600	0.009	3.373	0.378-30.076	0.276
Heart rate	1.046	1.023-1.069	< 0.001	1.009	0.971-1.048	0.649
Hypoalbuminemia	3.776	1.393-10.235	0.009	5.639	1.503-21.156	0.010
LDL-C	0.345	0.164-0.724	0.005	0.437	0.190-1.006	0.437
Total bilirubin	1.033	1.002-1.065	0.034	1.029	0.990-1.069	0.143
Indirect bilirubin	1.040	1.003-1.079	0.033	1.053	1.004-1.104	0.034
AST(per 10 IU/L)	1.011	1.005-1.018	0.001	1.027	0.997-1.058	0.776
LDH (per 10 IU/L)	1.010	1.005-1.014	<0.001	1.005	0.995-1.016	0.340
CK (per 100 IU/L)	1.013	1.005-1.022	0.001	1.014	0.981-1.048	0.404
CK-MB (per 10 IU/L)	1.076	1.027-1.127	0.002	0.913	0.732-1.138	0.417
Urea nitrogen	1.084	1.059-1.110	< 0.001	1.067	1.017-1.118	0.007
Cr (per 10 umol/L)	1.030	1.017-1.044	< 0.001	1.033	1.005-1.062	0.021
eGFR (per 10 mL/min)	1.014	1.008-1.020	< 0.001	1.015	1.002-1.028	0.024
Uric acid	1.006	1.003-1.009	< 0.001	1.005	1.000-1.010	0.043
Types of treatment			< 0.001			0.037
Medication	1			1		
Endovascular therapy	0.133	0.052-0.337	< 0.001	0.227	0.071-0.728	0.013
Surgery	0.393	0.052-2.955	0.364	0.246	0.015-4.154	0.331

sTIPS, the Simplified Thrombo-Inflammatory Prognostic Score; HR, hazard ratio; CI, confidence interval

## DISCUSSION

The present study indicated that a higher sTIPS correlated with a more complex baseline clinical patient risk profile, and that in-hospital mortality occurred more frequently in the group with higher sTIPS. When including sTIPS in a multivariable Cox regression model after adjusting for confounding factors, an elevated sTIPS was the most significant determinant of in-hospital mortality. Thus, our results suggest that the sTIPS may be able to stratify type B AAD patients based on mortality risk, in the early phase of admission.

AAD is characterized by intimal tear and surging of blood into the media layer of the artery, leading to the formation of a false lumen and subsequent systemic inflammatory responses [12]. Inflammation plays an important role in the development of AAD, and inflammatory cells such as lymphocytes and macrophages have been detected in medial degeneration [12, 13]. Furthermore, AAD patients with higher inflammatory cell activity in the aortic wall seemed to have a high risk for disease progression and poorer prognosis in the acute phase [14]. Moreover, several inflammatory markers such as interleukin-6 (IL-6), C-reactive protein (CRP), matrix metalloproteinases-9 (MMP-9), and WBC at admission were reportedly associated with the severity of systemic inflammatory response and adverse outcomes in patients with type A AAD [13, 15-19]. Similarly, high WBC at admission, platelet to lymphocyte ratio, and neutrophil to lymphocyte ratio were correlated with increased in-hospital mortality risk for type B AAD [20-22]. In contrast to studies reporting positive associations between CRP level at admission and risk of death in type A AAD, some studies have failed to find associations between baseline CRP and mortality risk in type B AAD patients [23, 24]. With respect to WBC, the predictive value for mortality in patients with AAD was not consistent [23, 25]. Regarding some other biomarkers such as cardiac troponin level at admission, there are conflicting reports about their accuracy to predict in-hospital mortality for patients with type A AAD [19, 26]. It appears that predicting adverse outcome in patients with type B AAD, based on biomarkers at admission, is more difficult compared to predicting for type A AAD. The association of these inflammatory biomarkers might be significantly affected by a time-dose expression curve or time-dependent changes, and thus their clinical value might be greatly compromised. A single biomarker might therefore only occasionally predict the prognosis of type B AAD.

Thrombosis is associated with the pathogenesis of AAD [27, 28]. Platelets play an important role in thrombosis, and are the first cellular components that adhere to the damaged vessel walls, followed by leukocytes, after an aortic intimal tear. This forms a thrombosis in the false lumen and modulates inflammatory activity [7, 13, 27]. Platelet activity is a predictor for thrombotic burden [29], and platelet indices at admission including PC, MPV, and MPV/PC ratio are useful markers for estimating platelet activity; MPV/PC ratio has been reported to be a better marker than MPV or PC alone [30, 31]. A previous study determined that PC was independently associated with in-hospital mortality in type A AAD, and among those undergoing surgical intervention [32].

Thrombocytopenia at admission was also a predictor of late mortality (50.8 months) in type B AAD by Cox proportional-hazards analysis in a small cohort study of 77 patients [33]. Our previous study indicated that an elevated MPV/PC ratio at admission was a strong independent predictor for in-hospital complications and long-term mortality (3.5 years) in patients with type A AAD [34]. Further, type A AAD patients with D-dimer at admission above 20 mg/mL had higher in-hospital mortality, but the predictive value became invalid after surgical intervention [25]. However, some studies did not find any association between PC or D-dimer values at admission and mortality in type B AAD patients [20, 35]. Given the inconsistent results, it remains unclear whether PC, D-dimer, or other markers of thrombosis at admission are predictive of in-hospital complications and long-term mortality among type B AAD patients.

Thrombo-inflammatory status can reflect the nature and severity of cardiovascular disease, and could be associated with other pathophysiological processes [9]. However, neither inflammatory nor thrombotic markers alone, might be particularly predictive of AAD; thus, a multi-biomarker strategy that combines biomarkers across patho-biological axes of inflammation and thrombosis provides incremental prognostic information for prediction of AAD [8, 10, 36]. To verify the hypothesis, we combined WBC (an inflammatory biomarker) and MPV/PC ratio (a thrombotic biomarker) at admission, into a novel and simplified thrombo-inflammatory prognostic score, and found that this score was independently predictive of in-hospital complications and long-term mortality in patients with type A AAD with higher sensitivity and specificity compared to WBC or MPV/PC ratio alone [10]. In the present study, we found that WBC or MPV/PC ratio alone were not independent predictors for in-hospital mortality in patients with type B AAD. However, when sTIPS was included in the multivariable Cox model, it could stratify high-risk patients with type B AAD in the hospital independently. Therefore, sTIPS might be a simple and effective prognostic evaluation tool combining both inflammatory and thrombotic biomarkers, and might be more practical for clinicians to identify high-risk patients with type B AAD, offering higher sensitivity and specificity, especially in the emergency room.

Recent studies revealed that false lumen circumferential extent, maximum aortic diameter, and false lumen thrombosis, were also helpful in identifying high-risk patients with type B AAD [37]. However, the anatomy of aortic dissection often changes during the course of type B AAD, and even after surgery and endovascular therapy. Our previous study showed that residual patent false lumen is independently associated with poor long-term survival in AAD. However, only type B AAD patients with partial false lumen thrombosis had an increased late mortality risk; this association was not found in type A AAD after surgery [7]. The sTIPS score predicted mortality and reflected systemic thrombo-inflammatory status irrespective of various therapies, and was more stable than CT markers. Therefore, a more accurate prognosis might be achieved by adding sTIPS to the current model.

## LIMITATIONS

Our analysis has several limitations. First, this study was a retrospective single-center cohort study, and many confounding factors might have affected the outcomes. In addition, we did not follow up with the patients, and could not investigate the association between sTIPS and long-term outcomes in type B AAD. Second, we recorded the WBC and MPV/PC ratio at admission, but the changes in these markers at different time points were not recorded; a series of measurements at different time points during hospitalization might be more valuable for evaluation of the association between sTIPS and prognosis of type B AAD. Third, we did not measure other inflammatory and thrombotic biomarkers, such as IL-6, MMP-9, CRP, and D-dimer, and investigate the relationship between these biomarkers and adverse outcomes in type B AAD. Fourth, we did not have data on the CT anatomical parameters of the included patients; hence, we could not compare the predictive value of anatomical parameters for in-hospital mortality of patients with type B AAD. Therefore, prospective, large-scale multicenter studies are required to confirm our results.

## CONCLUSIONS

In conclusion, the sTIPS is a useful score reflecting the overall inflammatory-thrombotic status, and was a powerful predictor of in-hospital mortality among patients with type B AAD, irrespective of the type of therapy. The sTIPS may be therefore able to risk-stratify type B AAD patients at admission for in-hospital outcomes such as mortality in the early phase.

## References

[1] Nienaber CA, Eagle KA (2003). Aortic dissection: new frontiers in diagnosis and management: Part I: from etiology to diagnostic strategies. Circulation.

[2] Tolenaar JL, Froehlich W, Jonker FH, Upchurch GR, Rampoldi V, Tsai TT, Bossone E, Evangelista A, O’Gara P, Pape L, Montgomery D, Isselbacher EM, Nienaber CA (2014). Predicting in-hospital mortality in acute type B aortic dissection: evidence from International Registry of Acute Aortic Dissection. Circulation.

[3] Liang NL, Genovese EA, Al-Khoury GE, Hager ES, Makaroun MS, Singh MJ (2017). Effects of Gender Differences on Short-term Outcomes in Patients with Type B Aortic Dissection. Ann Vasc Surg.

[4] Ko T, Higashitani M, Sato A, Uemura Y, Norimatsu T, Mahara K, Takamisawa I, Seki A, Shimizu J, Tobaru T, Aramoto H, Iguchi N, Fukui T (2015). Impact of Acute Kidney Injury on Early to Long-Term Outcomes in Patients Who Underwent Surgery for Type A Acute Aortic Dissection. Am J Cardiol.

[5] Zeng R, Li D, Deng L, He Y, Sun X, Wan Z, Nie H, Du X, Cao Y, Zeng Z (2016). Hypoalbuminemia predicts clinical outcome in patients with type B acute aortic dissection after endovascular therapy. Am J Emerg Med.

[6] Kudo T, Mikamo A, Kurazumi H, Suzuki R, Morikage N, Hamano K (2014). Predictors of late aortic events after Stanford type B acute aortic dissection. J Thorac Cardiovasc Surg.

[7] Li D, Ye L, He Y, Cao X, Liu J, Zhong W, Cao L, Zeng R, Zeng Z, Wan Z, Cao Y (2016). False Lumen Status in Patients With Acute Aortic Dissection: A Systematic Review and Meta-Analysis. J Am Heart Assoc.

[8] Li DZ, Yu J, Du RS, Zeng R, Zeng Z (2016). Thrombo-inflammatory status and prognosis of acute type A aortic dissection. Herz.

[9] Nagareddy P, Smyth SS (2013). Inflammation and thrombosis in cardiovascular disease. Curr Opin Hematol.

[10] Li DZ, Li XM, Sun HP, Yang YN, Ma YT, Qu YY, Ma X, Liu F (2015). A novel simplified thrombo-inflammatory prognostic score for predicting in-hospital complications and long-term mortality in patients with type A acute aortic dissection: a prospective cohort study. Eur Heart J Suppl.

[11] Levey AS, Coresh J, Greene T, Stevens LA, Zhang YL, Hendriksen S, Kusek JW, Van Lente F (2006). Using standardized serum creatinine values in the modification of diet in renal disease study equation for estimating glomerular filtration rate. Ann Intern Med.

[12] Luo F, Zhou XL, Li JJ, Hui RT (2009). Inflammatory response is associated with aortic dissection. Ageing Res Rev.

[13] Kurihara T, Shimizu-Hirota R, Shimoda M, Adachi T, Shimizu H, Weiss SJ, Itoh H, Hori S, Aikawa N, Okada Y (2012). Neutrophil-derived matrix metalloproteinase 9 triggers acute aortic dissection. Circulation.

[14] Kuehl H, Eggebrecht H, Boes T, Antoch G, Rosenbaum S, Ladd S, Bockisch A, Barkhausen J, Erbel R (2008). Detection of inflammation in patients with acute aortic syndrome: comparison of FDG-PET/CT imaging and serological markers of inflammation. Heart.

[15] Fan X, Huang B, Lu H, Zhao Z, Lu Z, Yang Y, Zhang S, Hui R (2015). Impact of Admission White Blood Cell Count on Short- and Long-term Mortality in Patients With Type A Acute Aortic Dissection: An Observational Study. Medicine.

[16] Wen D, Wu HY, Jiang XJ, Zhang HM, Zhou XL, Li JJ, Hui RT (2011). Role of plasma C-reactive protein and white blood cell count in predicting in-hospital clinical events of acute type A aortic dissection. Chin Med J.

[17] Gu J, Hu J, Zhang HW, Xiao ZH, Fang Z, Qian H, Zhong MH, Guo YQ, Zhang EY, Shi YK, Meng W (2015). Time-dependent changes of plasma inflammatory biomarkers in type A aortic dissection patients without optimal medical management. J Cardiothorac Surg.

[18] Wen D, Zhou XL, Li JJ, Luo F, Zhang L, Gao LG, Wang LP, Song L, Sun K, Zou YB, Zhang CN, Hui RT (2012). Plasma concentrations of interleukin-6, C-reactive protein, tumor necrosis factor-alpha and matrix metalloproteinase-9 in aortic dissection. Clin Chim Acta.

[19] Vrsalovic M, Zeljkovic I, Presecki AV, Pintaric H, Kruslin B (2015). C-reactive protein, not cardiac troponin T, improves risk prediction in hypertensives with type A aortic dissection.

[20] Chen ZR, Huang B, Lu HS, Zhao ZH, Hui RT, Yang YM, Fan XH (2017). Admission white blood cell count predicts short-term clinical outcomes in patients with uncomplicated Stanford type B acute aortic dissection. J Geriatr Cardiol.

[21] Du R, Li D, Yu J, Ma Y, Zhang Q, Zeng Z, Zeng R (2017). Association of platelet to lymphocyte ratio and risk of in-hospital mortality in patients with type B acute aortic dissection. Am J Emerg Med.

[22] Sbarouni E, Georgiadou P, Analitis A, Voudris V (2015). High neutrophil to lymphocyte ratio in type A acute aortic dissection facilitates diagnosis and predicts worse outcome. Expert Rev Mol Diagn.

[23] Okina N, Ohuchida M, Takeuchi T, Fujiyama T, Satoh A, Sakamoto T, Adachi H, Imaizumi T (2013). Utility of measuring C-reactive protein for prediction of in-hospital events in patients with acute aortic dissection. Heart Vessels.

[24] Sakakura K, Kubo N, Ako J, Wada H, Fujiwara N, Funayama H, Ikeda N, Nakamura T, Sugawara Y, Yasu T, Kawakami M, Momomura S (2010). Peak C-reactive protein level predicts long-term outcomes in type B acute aortic dissection. Hypertension.

[25] Tian L, Fan X, Zhu J, Liang Y, Li J, Yang Y (2014). Plasma D-dimer and in-hospital mortality in patients with Stanford type A acute aortic dissection. Blood Coagul Fibrinolysis.

[26] Vrsalovic M (2016). Prognostic effect of cardiac troponin elevation in acute aortic dissection: A meta-analysis. Int J Cardiol.

[27] Owens AP, Edwards TL, Antoniak S, Geddings JE, Jahangir E, Wei WQ, Denny JC, Boulaftali Y, Bergmeier W, Daugherty A, Sampson UK, Mackman N (2015). Platelet Inhibitors Reduce Rupture in a Mouse Model of Established Abdominal Aortic Aneurysm. Arterioscler Thromb Vasc Biol.

[28] Zhang S, Qian H, Yang Q, Hu J, Gan C, Meng W (2015). Relationship between the extent of dissection and platelet activation in acute aortic dissection. J Cardiothorac Surg.

[29] Alexopoulos D, Xanthopoulou I, Tsigkas G, Damelou A, Theodoropoulos KC, Makris G, Gizas V, Kassimis G, Davlouros P, Hahalis G (2013). Intrinsic platelet reactivity and thrombus burden in patients with ST-elevation myocardial infarction. Thromb Res.

[30] Azab B, Torbey E, Singh J, Akerman M, Khoueiry G, McGinn JT, Widmann WD, Lafferty J (2011). Mean platelet volume/platelet count ratio as a predictor of long-term mortality after non-ST-elevation myocardial infarction. Platelets.

[31] Gao XM, Moore XL, Liu Y, Wang XY, Han LP, Su Y, Tsai A, Xu Q, Zhang M, Lambert GW, Kiriazis H, Gao W, Dart AM (2016). Splenic release of platelets contributes to increased circulating platelet size and inflammation after myocardial infarction. Clin Sci.

[32] Huang B, Tian L, Fan X, Zhu J, Liang Y, Yang Y (2014). Low admission platelet counts predicts increased risk of in-hospital mortality in patients with type A acute aortic dissection. Int J Cardiol.

[33] Delsart P, Beregi JP, Devos P, Haulon S, Midulla M, Mounier-Vehier C (2014). Thrombocytopenia: an early marker of late mortality in type B aortic dissection. Heart Vessels.

[34] Li DZ, Chen QJ, Sun HP, Zeng R, Zeng Z, Gao XM, Ma YT, Yang YN (2016). Mean platelet volume to platelet count ratio predicts in-hospital complications and long-term mortality in type A acute aortic dissection. Blood Coagul Fibrinolysis.

[35] Kitada S, Akutsu K, Tamori Y, Yoshimuta T, Hashimoto H, Takeshita S (2008). Usefulness of fibrinogen/fibrin degradation product to predict poor one-year outcome of medically treated patients with acute type B aortic dissection. Am J Cardiol.

[36] Wang TJ (2011). Assessing the role of circulating, genetic, and imaging biomarkers in cardiovascular risk prediction. Circulation.

[37] Sailer AM, van Kuijk SM, Nelemans PJ, Chin AS, Kino A, Huininga M, Schmidt J, Mistelbauer G, Baumler K, Chiu P, Fischbein MP, Dake MD, Miller DC (2017). Computed Tomography Imaging Features in Acute Uncomplicated Stanford Type-B Aortic Dissection Predict Late Adverse Events. Circ Cardiovasc Imaging.

